# DNA methylation patterns in bladder cancer and washing cell sediments: a perspective for tumor recurrence detection

**DOI:** 10.1186/1471-2407-8-238

**Published:** 2008-08-14

**Authors:** Priscilla D Negraes, Francine P Favaro, João Lauro V Camargo, Maria Luiza CS Oliveira, José Goldberg, Cláudia A Rainho, Daisy MF Salvadori

**Affiliations:** 1 Department of Genetics, Biosciences Institute, UNESP, Sao Paulo State University, Botucatu, Sao Paulo, Brazil; 2 Department of Pathology, Botucatu Medical School, UNESP, Sao Paulo State University, Botucatu, Sao Paulo, Brazil; 3 Department of Urology, Botucatu Medical School, UNESP, Sao Paulo State University, Botucatu, Sao Paulo, Brazil. CEP 18618-000

## Abstract

**Background:**

Epigenetic alterations are a hallmark of human cancer. In this study, we aimed to investigate whether aberrant DNA methylation of cancer-associated genes is related to urinary bladder cancer recurrence.

**Methods:**

A set of 4 genes, including *CDH1 *(E-cadherin), *SFN *(stratifin), *RARB *(retinoic acid receptor, beta) and *RASSF1A *(Ras association (RalGDS/AF-6) domain family 1), had their methylation patterns evaluated by MSP (Methylation-Specific Polymerase Chain Reaction) analysis in 49 fresh urinary bladder carcinoma tissues (including 14 cases paired with adjacent normal bladder epithelium, 3 squamous cell carcinomas and 2 adenocarcinomas) and 24 cell sediment samples from bladder washings of patients classified as cancer-free by cytological analysis (control group). A third set of samples included 39 archived tumor fragments and 23 matched washouts from 20 urinary bladder cancer patients in post-surgical monitoring. After genomic DNA isolation and sodium bisulfite modification, methylation patterns were determined and correlated with standard clinic-histopathological parameters.

**Results:**

*CDH1 *and *SFN *genes were methylated at high frequencies in bladder cancer as well as in paired normal adjacent tissue and exfoliated cells from cancer-free patients. Although no statistically significant differences were found between *RARB *and *RASSF1A *methylation and the clinical and histopathological parameters in bladder cancer, a sensitivity of 95% and a specificity of 71% were observed for *RARB *methylation (Fisher's Exact test (p < 0.0001; OR = 48.89) and, 58% and 17% (p < 0.05; OR = 0.29) for *RASSF1A *gene, respectively, in relation to the control group.

**Conclusion:**

Indistinct DNA hypermethylation of *CDH1 *and *SFN *genes between tumoral and normal urinary bladder samples suggests that these epigenetic features are not suitable biomarkers for urinary bladder cancer. However, *RARB *and *RASSF1A *gene methylation appears to be an initial event in urinary bladder carcinogenesis and should be considered as defining a panel of differentially methylated genes in this neoplasia in order to maximize the diagnostic coverage of epigenetic markers, especially in studies aiming at early recurrence detection.

## Background

Urinary bladder cancer is the fourth most common malignancy in the Western world, with a male:female ratio of nearly four to one and a median age at diagnosis between 65 and 70 years [1]. Histologically, 90% to 95% of malignant bladder tumors are urothelial carcinoma (UC), formerly designated transitional cell carcinoma (TCC) [2]. Although more than 70% of the lesions are detected as non-invasive papillary carcinomas, which commonly recur, a poor prognosis is related to tumors that are already invasive at diagnosis (~20%) [3]. After transurethral resection of superficial bladder cancer, periodic cystoscopic monitoring is performed for early recurrence detection, with some cases requiring intravesical prophylactic instillation chemotherapy. Muscle invasive disease calls for more aggressive treatment, often consisting of radical cystectomy and bladder substitution [4].

At present, conventional diagnosis for urinary bladder cancer is based on morphological, histological and pathological features. These criteria provide essential prognostic information, but show insufficient power to precisely predict patient outcome. The need for accurate predictive markers has led to the search for molecular markers in bladder cancer patients [5]. The use of genetic and epigenetic alterations for the early detection of bladder cancer is promising because it is believed that some molecular events occur at the beginning of the carcinogenesis process. Thus, molecular diagnosis may allow detection before clinical or radiographic manifestations. In this context, a sensitive and specific noninvasive test could prescreen patients with clinical symptoms as well as those at high risk, and would also be useful in monitoring patients post-surgically for early detection of recurrence.

DNA-, RNA-based or/and immunohistochemical methods have been applied to identify new tumor markers or to estimate risk of tumor progression in UC. Several DNA alterations have been described in bladder cancer, such as allele losses or deletions [6], gene amplifications [7], DNA mutations [8] and microsatellite instabilities [9]. Furthermore, aberrant DNA methylation patterns have been recognized as common epigenetic changes in human cancer and are already detected in early cancer stages [10]. DNA methylation occurs on cytosine residues located at the 5' position of guanines in CpG dinucleotides [11]. Its distribution on the mammalian genome is not random and is especially important in CpG-rich areas, also called CpG islands. The promoter region of actively transcribing genes is frequently rich in this dinucleotide sequence, almost always unmethylated [12].

Dense DNA methylation in CpG islands of growth-regulating gene promoter regions is now recognized as a common alternative mechanism for gene inactivation in human cancer, an event as important as somatic mutations in coding regions of tumor suppressor genes (TSG) [13]. Usually both genetic and epigenetic events represent complementary hits involved in TSG inactivation [14]. A large number of studies have shown that loci of epigenetically inactivated TSG generally coincide with overlapping regions of allelic losses in human cancer, including UC [6,15-19]. In fact, loss of heterozygosity (LOH) assays have been widely used as indirect approaches in the search for a new TSG [20,21]. In the last few years, genetic studies have indicated that allelic loss in many distinct chromosomal regions, including 1p, 3p, and 16q, are associated with UC tumorigenesis [17-19,21]. It is important to notice that *RASSF1A *(Ras association (RalGDS/AF-6) domain family 1) and *RARB *(retinoic acid receptor, beta) mapped at 3p (3p21.3 and 3p24, respectively), *SFN *(stratifin, also known as 14-3-3σ) located at 1p35.3, and *CDH1 *(cadherin 1, type 1, E-cadherin [epithelial]) at 16q22.1, are epigenetically silenced TSGs located at loci that overlap with LOH minimal regions in human cancer.

RASSF1A protein probably modulates some of the growth inhibitory responses mediated by Ras, although its interaction with activated Ras remains unclear. This gene is considered a bona fide tumor suppressor epigenetically inactivated during human carcinogenesis, whose hypermethylation has also been reported in UC [22-30]. The *RARB *gene is a member of the thyroid-steroid hormone receptor superfamily of nuclear transcriptional regulators that binds retinoic acid (the biologically active form of vitamin A), and also mediates cellular signaling during embryonic morphogenesis, cell growth, and differentiation [31]. Retinoic acids exhibit tumor-suppressor activity due to their antiproliferative and apoptosis-inducing effects [32]. *RARB *has also presented high methylation frequencies in urinary bladder tumors (varying from 15% to 93%) [15,22,24,25,30].

Initially, it was suggested that loss of stratifin expression could contribute to malignant transformation by disabling the cell cycle arrest at the G2 checkpoint, allowing the accumulation of genetic defects [33]. Subsequently, the down-regulation of *SFN *gene in various human cancers was generally attributed to the hypermethylation of its CpG island. To the best of our knowledge, there is only one previous study addressing *SFN *hypermethylation in UC where the highest frequency was found for squamous cell carcinomas irrespective of their grade of cellular malignancy (80%). Furthermore, the authors found hypermethylation of 57.1% for high grade, high stage nonpapillary TCC; 28.6% for low grade, low stage papillary TCC; and 28% for undifferentiated small cell carcinomas, the lowest rate [34].

The *CDH1 *gene encodes for a calcium-dependent cell-cell adhesion glycoprotein, whose loss of function may contribute to cancer progression by increasing proliferation, invasion, or metastasis [35]. These findings suggest that *CDH1 *is a tumor- and invasion-suppressor gene [36]. Its relation to urinary bladder carcinogenesis was demonstrated through the observation of altered expression due to epigenetic changes in many studies, ranging from 9.5% hypermethylation [29] up to 87% in TCCs [37]. However, some studies have also evaluated TCCs, both in squamous cells and *in situ *carcinoma, and have shown a variable spectrum of hypermethylation for the same gene [15,22,24,25,27,30,38-40].

In an effort to identify a possible association among epigenetic changes, urinary bladder cancer prognosis and early-recurrence, we analyzed the methylation pattern of *CDH1, RARB, SFN *and *RASSF1A *genes in 54 fresh samples of urinary bladder cancer, 14 of which were paired with tumor-adjacent normal urothelium; 39 paraffin-embedded UC primary tumor and/or recurrence matched with 23 bladder washing sediments obtained from 20 patients under post-surgical monitoring. In addition, we analyzed a hospital-based control group consisting of 24 bladder washings from patients that reported urological complaints, but without any bladder tumor history and showing negative cytology for tumor cell presence.

## Methods

### Sample collection and DNA extraction

Methylation patterns of *RASSF1A, RARB, SFN*, and *CDH1 *genes were determined in two cell lineages, 5637 and T24, derived from non-invasive and invasive high-grade UC, respectively. Fresh samples of tumoral urinary bladder tissues were obtained from 54 patients (44 males and 10 females; median age of 67.85 years, ranging from 40 to 90 years) who underwent surgical treatment at Amaral Carvalho Hospital, Jaú, SP, Brazil. Patients were recruited consecutively on the basis of tissue availability. Treatment for each patient consisted of initial endoscopic tumor resection and subsequent radical cystectomy for those with muscle invasive disease. Non-muscle invasive tumors underwent intravesical bacillus Calmette-Guerin (BCG) therapy. Normal adjacent tissue samples were also collected from each case. A tumor fragment and the matched normal adjacent tissue were fixed in formalin and embedded in paraffin. The corresponding hematoxylin-eosin-stained sections were evaluated by the same pathologist (JLVC) to determine tumor type, grade and growth pattern. Samples were trimmed to maximize the quantity of target tissue and only fragments with more than 70% neoplastic cells were used for DNA extraction. After this evaluation, only 14 normal adjacent tissue samples exhibited an epithelial layer. Tumors were staged according to the 1998 WHO-ISUP classification [41].

A control group included 24 urinary bladder washings from patients that reported urological complaints, but without any bladder tumor history and showing negative cytology for tumor cell presence in the same bladder washing cell samples.

In addition, 20 patients in post-surgical monitoring, who underwent cytology analysis to detect tumor recurrence, were recruited at the Department of Urology from Botucatu Medical School, UNESP – Sao Paulo State University, Brazil. From this group was collected a total of 23 urinary bladder washings matched with 39 UC samples obtained from the Department of Pathology archive. Among these 20 patients 9 presented recurrent tumors (analysis until seven biopsies had been collected at distinct times) and 11 were primary tumors.

Genomic DNA from fresh bladder tissues, paraffin-embedded samples and washout cell sediments were obtained by standard sodium dodecyl sulfate/proteinase K digestion, followed by phenol/chloroform extraction and ethanol precipitation.

All samples were collected after patients or their relatives had provided informed consent. Approval for research on human subjects was obtained from the respective ethic committees of both institutions and by the National Research Ethics Committee (CONEP 9382) Brasilia, DF, Brazil.

### Bisulfite treatment and Methylation-Specific Polymerase Chain Reaction (MSP)

The conversion of DNA by sodium bisulfite was performed using an established protocol [42] with modifications. Initially, genomic DNA was denatured with 3 M NaOH at 40°C for 15 min (final concentration of 0.3 M NaOH). The urea/bisulfite and hydroquinone solution (freshly prepared, pH 5.0) were then added to the denatured DNA to yield final concentrations of 5.36 M, 3.44 M, and 0.5 mM, respectively, followed by 20 cycles of incubation at 55°C for 15 min followed by denaturation at 95°C for 30 sec in a PTC200 Peltier Termal Cycler (MJ Research, Madison, USA). DNA was purified with the Wizard DNA Clean-UP System (Promega. Madison, WI, USA), and DNA modification was completed by the addition of 5.0 μl of NaOH 3 M at room temperature for 15 min. Precipitation was carried out through the addition of 30 μl of ammonium acetate 5 M (pH 7.0), 350 μl of ethanol and 1 μl of glycogen (20 μg/uL) (Invitrogen Life Technologies, Carlsbad, CA, USA). The bisulfite-modified DNA was resuspended in 20 μl of sterile water and stored at -20°C.

The methylation pattern of promoter regions for *CDH1, RARB, SFN *and *RASSF1A *genes was evaluated by a MSP approach. For each gene, previously described primers specific to the methylated and unmethylated sequences were used [33,43-45]. DNA from lymphocytes of healthy volunteers treated with *SssI *methyltransferase (New England Biolabs, Beverly, MA, USA) and then subjected to bisulfite modification was used as positive controls for methylated alleles. The reaction was performed in a total volume of 50 μl containing 10 μg of genomic DNA, 10 U of *SssI *methylase, 160 mM of S-adenosyl-metionina, 50 mM of NaCl, 10 mM of Tris-HCl, 10 mM of MgCl_2_, 1 mM of DTT pH 7.9, during 18 hours at 37°C.

Table 1 summarizes the oligonucleotide sequences, annealing temperature and product size for MSP analysis. To determine the methylation pattern within the CpG island in 5'UTR of the *CDH1 *gene, a nested-PCR approach was used as previously described in detail [46].

**Table 1:** Oligonucleotide sequences, annealing temperatures, and product size for MSP analysis.

**Gene (MS)**	**Primers 5'- 3'**	Position of interrogated CpGs*	**Ta (°C)**	**Product size (bp)**	**Ref**.
***CDH1***					
Methylated allele	GTAGTTACGTATTTATTTTTAGTGGCGTC (F)	-14; 4; 7	53	112	[43]
	CGAATACGT CGAATCGAACCG (R)	68; 73; 78; 82; 88			
Unmethylated allele	TGGTTGTAGTTATGTATTTATTTTTAGTGGTGTT (F)		53	120	
	ACACCAATACAACAAATCAAACCAAA (R)				
***RARB***					
Methylated allele	GAACGCGAGCGATTCGAGT (F)	111; 113; 117; 122	55	158	[44]
	GACCAATCCAACCGAAACG (R)	231; 236; 249			
Unmethylated allele	GGATTGGGATGTTGAGAATG (F)		55	143	
	CAACCAATCCAACCAAAACAA (R)				
***SFN***					
Methylated allele	GGTAGTTTTTATGAAAGGCGTC (F)	153; 156	56	106	[33]
	CCTCTAACCGCCCACCACG (R)	219; 228			
Unmethylated allele	ATGGTAGTTTTTATGAAAGGTGTT (F)		56	104	
	CCCTCTAACCACCCACCACA (R)				
***RASSF1A***					
Methylated allele	GGGTTTTGCGAGAGCGCG (F)	-66; -60; -58	55	169	[45]
	GCTAACAAACGCGAACCG (R)	77; 82; 84; 94			
Unmethylated allele	GGTTTTGTGAGAGTGTGTTTAG (F)		55	169	
	CACTAACAAACACAAACCAAAC (R)				

MSP – Methylation-Specific Polymerase Chain Reaction; MS – methylation status; * in relation to transcription start site; Ta – Annealing temperature; bp – base pair; F – forward primer; R – reverse primer.

One-step MSP was performed to detect the methylation pattern of *RARB, SFN *and *RASSF1A *genes, using specific primers for the methylated and unmethylated sequences in distinct reactions, accomplished in a total volume of 25 μl containing 0.25 μM of each primer, 200 μM of each dNTP, 15 mM Tris-HCl, pH 8.0, 50 mM KCl, 1U of AmpliTaq Gold (Applied Biosystems, Foster City, CA, USA) and 3 mM MgCl_2 _for *RARB *and *RASSF1A*, and 2.5 mM MgCl_2 _for *SFN*.

The amplified products were visualized after electrophoresis in 6% polyacrylamide gel and silver nitrate staining [47]. Water blanks were included in each assay.

### Statistical analysis

Descriptive mean and percentage statistics were used to summarize patient data and gene hypermethylation status. The presence of methylation and characteristics including age, sex and clinico-histopathological parameters were evaluated using Odds Ratio (OR) with Confidence Interval (CI) of 95%. Pairwise associations followed dichotomous variables defined according to growth pattern (non-papillary *versus *papillary), differentiation grade (low *versus *high), tumor invasiveness (noninvasive *versus *invasive) [41], and presence or absence of tumoral recurrence. Potential associations on the presence of promoter methylation for each gene as well as the sensitivity and specificity of the assay for tumor recurrence were assessed using Fisher's Exact test with a 5% significance level. Correlations between cytology and hypermethylation of bladder washings were considered to assess the relative hazar of recurrence. All statistical evaluations were performed using a computer-assisted program (SPSS – Statistical Package for The Social Sciences v15.0, SPSS Inc.).

## Results

### MSP analysis in cell lineages

MSP analysis of DNA from 5637 and T24 cell lines evidenced hypermethylation at the *CDH1 *and *RASSF1A *gene promoter regions. None of them showed this pattern for the *RARB *gene. The methylation of *SFN *was observed in 5637 cells, but was not present in T24.

### MSP analysis in matched tumoral and adjacent bladder tissue samples

Fifty-four matched tumoral and adjacent tissue samples were collected. Remarkably, after histopathology, the presence of normal epithelial cell layer in normal adjacent biopsies was confirmed in only 14 pairs. Thus, MSP analysis was performed on 49 UCs obtained from unrelated patients, 14 of them matched with normal tissue samples, 3 squamous cell carcinomas and 2 adenocarcinomas. Table 2 summarizes the relevant clinical and histopathological characteristics in the group of 49 UC patients. On average, these patients received 30 months of follow-up monitoring. Twenty-five UC patients showed recurrence: 20 (40.8%) were recurrent at the moment of the study sample collection, since they had a positive history of previous UC confirmed by a histological diagnosis before the date of the most recent surgery (0.7 to 7.5 years); and 5 had their primary tumor evaluated, but exhibited recurrence within a short time period (Table 3).

**Table 2:** Clinical and histopathological data from patients with UC tumors.

**Clinical and histopathological features**	
**Patients, n**	49
**Sex, n (%)**	
Male	40 (81.6%)
Female	9 (18.4%)
Age, n (%)*	
≤ 60 years	9 (18.7%)
> 60 years	39 (81.3%)
**Growth pattern, n (%)**	
Papillary	33 (67.3%)
Non-papillary	16 (32.7%)
**Muscle invasion, n (%)**	
Noninvasive	30 (61.2%)
Invasive	19 (38,8%)

* 48 patients contributed to this information

**Table 3:** Clinical and histopathological prevalence parameters and DNA methylation pattern for *RARB *and *RASSF1A *genes in 49 fresh urinary bladder carcinoma tissues.

**Variable**	***RARB***		**OR (95% CI)**	***RASSF1A***		**OR (95% CI)**
	**N^(a)^**	**P^(a)^**		**N^(a)^**	**P^(a)^**	
**Age^#^**						
< 60 years	1	8	1.0 (ref)	6	3	1.0 (ref)
≥ 60 years	8	31	0.48 (0.05–4.45)	31	8	0.51 (0.10–2.52)
Sex*						
Female	3	6	1.0 (ref)	9	0	na *p*^(1) ^= 0,097
Male	6	34	2.83 (0.55–14.54)	29	11	
Growth pattern*^(b)^						
Non-papillary	2	7	1.0 (ref)	12	4	1.0 (ref)
Papillary	14	26	0.53 (0.09–2.90)	26	7	0.80 (0.19–3.29)
Differentiation grade*^(*b*)^						
Low	5	16	1.0 (ref)	18	3	1.0 (ref)
High	4	24	1.88 (0.44–8.07)	20	8	2.40 (0.55–10.46)
Muscle invasion*^(*b*)^						
Noninvasive	7	23	1.0 (ref)	24	6	1.0 (ref)
Invasive	2	17	2.59 (0.48–14.05)	14	5	1.43 (0.37–5.55)
Post-surgery recurrence*						
Absence	4^(0/20)^	22^(4/20)^	1.0 (ref)	19^(3/20)^	7^(1/20)^	1.0 (ref)
Presence	5^(1/20)^	18^(15/20)^	0.65 (0.15–2.80)	19^(13/20)^	4^(3/20)^	0.57 (0.14–2.27)

^#^Includes 48 samples; *Includes 49 samples; N – methylation negative; P – methylation positive; (a) patients that showed recurrence among those that were under recurrence at time of the tumor sample collection; (b) according to recommendations of WHO-ISUP 1998 [43]; OR – odds ratio; CI – confidence interval; ref – referent category; na – not applicable; (1) Fisher's Exact test (α = 0.05).

After genomic DNA treatment with sodium bisulfite and MSP analysis, both amplicons for unmethylated and methylated alleles, respectively, were detected in *CDH1 *and *SFN *gene target regions in all 49 fresh UC samples. Among histopathologically normal tumor-adjacent urinary bladder tissues, the same methylation pattern was found, except in one sample which showed only the unmethylated alleles for *SFN *gene.

**Figure 1:**
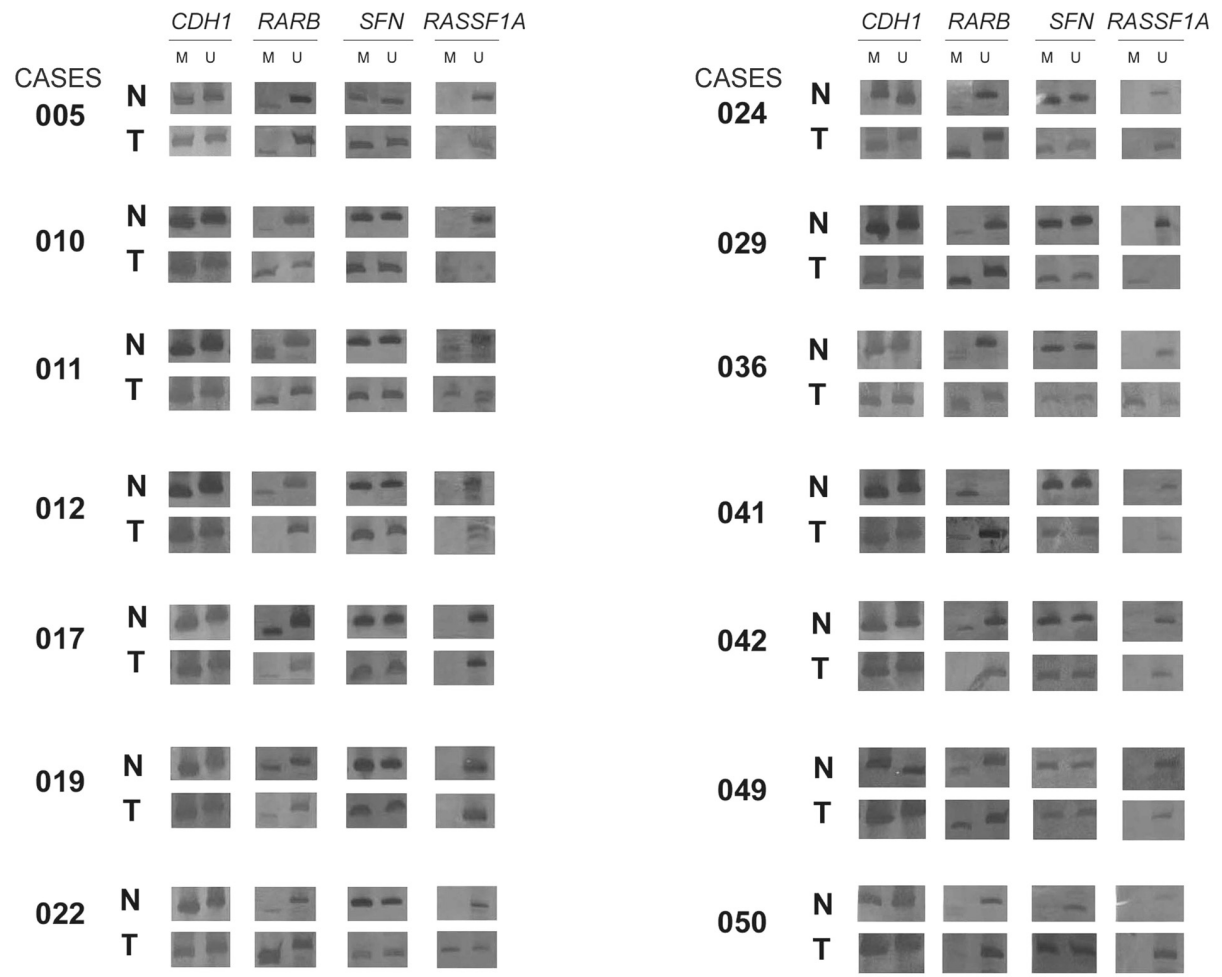
***CDH1, RARB, SFN *****and*****RASSF1A*****methylation patterns observed in fresh UC samples paired with normal adjacent urinary bladder tissue.** M – methylated allele; U – unmethylated allele; N – normal tissue; T – tumoral tissue.

*RARB *and *RASSF1A *hypermethylation were detected in 40 (81.6%) and 11 (22.5%) UC samples, respectively. The comparison of 14 matched normal and tumoral urinary bladder samples exhibited a concordant pattern for presence of *RARB *hypermethylation in 12 pairs; in two pairs (cases 12 and 42, Figure 1) *RARB *hypermethylation was restricted to normal tissue. Absence of *RASSF1A *hypermethylation was a common feature in 9 bladder tissue pairs. Only one case showed hypermethylation in both normal and tumoral tissues (case 11, Figure 1) and, in 3 matched samples (cases 22, 29 and 36, Figure 1) it was restricted to the tumor specimens. Adjusted ORs for these data as well as demographic, physician and histopathological parameters related to the *RARB *and *RASSF1A *DNA methylation patterns in 49 UC samples are shown in Table 3.

The adenocarcinomas analyzed showed hypermethylation for both, *RARB *and *RASSF1A *genes. Two of the squamous cell carcinomas showed the same methylation pattern for *RARB*, and one for *RASSF1A*.

### MSP analysis in exfoliated cells (bladder washings) in cancer-free controls and in bladder cancer patients

In the control group, of the 24 urinary bladder exfoliated cell samples submitted to cytological analysis from patients (median age of 61.4, ranging from 26 to 82 years) with negative diagnosis for cancer (four of them with cystitis), 2/23 (8.3%) and 8/16 (50%) exhibited *RARB *and *RASSF1A *hypermethylation, respectively. In addition, higher frequencies of methylation were detected for *CDH1 *(91.3%) and *SFN *(95.5%) genes. Compared to the results shown by the biopsy analysis of UCs, *RARB *had a sensitivity of 95% and specificity of 71% by Fisher's Exact test (p < 0.0001; OR = 48.89); for the same parameters, *RASSF1A *showed 58% and 17%, respectively (p < 0.05; OR = 0.29) (Table 4).

**Table 4:** Accuracy patterns in urinary bladder tumor biopsies and control group bladder washings (cell sediment samples from bladder washings of patients classified as cancer-free by cytological analysis).

**Sample**	**Accuracy patterns (%)**							
	
	***RARB***				***RASSF1A***			
	**OR**	**Sens**.	**Spec**.	**p^(1)^**	**OR**	**Sens**.	**Spec**.	**p^(1)^**
	**(95% CI)**				**(95% CI)**			
**Control group**	1.0 (ref)	95%	71%	0.0001	1.0 (ref)	58%	17%	0.005
**Tumor biopsies**	48.89 (9.69–246.67)				0.29 (0.09–0.95)			

Sens. – Sensitivity; Spec. – Specificity; ref – referent category; CI – confidence interval; (1) Fisher's Exact test (α = 0.05)

**Figure 2:**
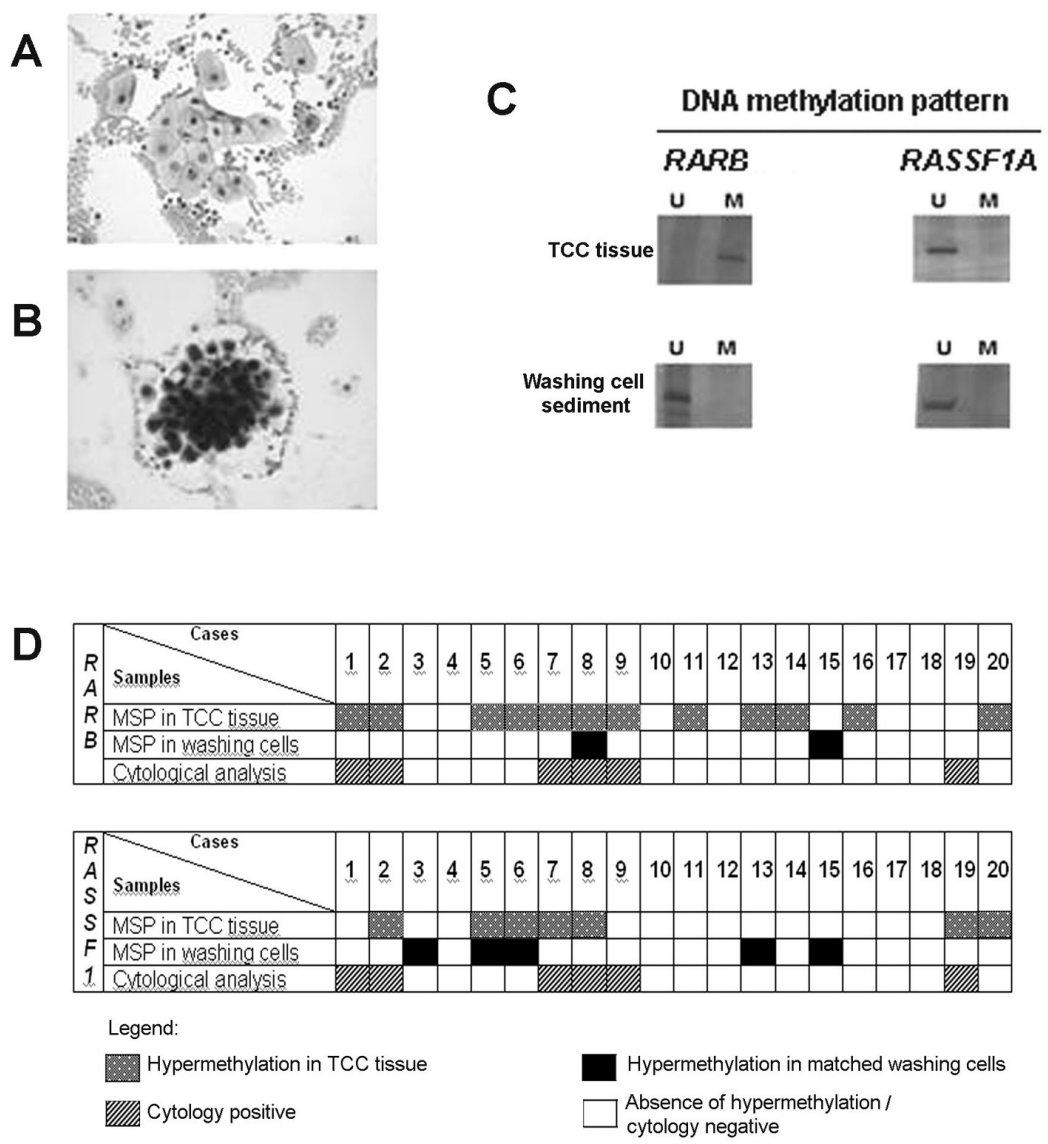
**A) Cytological analysis of bladder washing sediment negative for the presence of tumor cells (case 11).** B) Positive cytology illustrating a tumor recurrent case during the post-surgical monitoring. × 400, Giemsa staining. C) Comparative MSP results from case 11 in tumor tissue (TCC) and in the exfoliated cells from the correspondent bladder washing for *RARB *and *RASSF1A *genes. M – methylated allele; U – unmethylated allele. D) Distribution of MSP results among the third set of samples including 39 archived tumor fragments and 23 matched washouts from 20 urinary bladder cancer patients in post-surgical monitoring and comparison with the cytological analysis.

In the third sample set, hypermethylation identified in tumor DNA from archival UC samples was used as a molecular tag to predict tumor recurrence in the corresponding DNA obtained from cells of urinary bladder washings from UC patients under post-surgical monitoring. The comparative analysis between MSP from washout cells and corresponding primary and/or recurrence tumor sample was done for *RARB *and *RASSF1A *genes, including 23 urinary bladder washings and 39 paraffin-embedded UC samples from 20 patients (median age of 68.65, ranging from 42 to 84 years) (Figure 2). Among 39 tissue samples, *RARB *hypermethylation was identified in 14/38 fragments (36.8%) analyzed. Twelve patients showed at least one tumor fragment hypermethylated for *RARB *gene. Due to limited tissue volume, *RASSF1A *gene was analyzed in a subset of 15 fragments from 11 patients, with methylation detected in 9/15 of them (60%); these hypermethylated tumors were from 7 patients.

Patients showing at least one hypermethylated tumor fragment for *RARB *and/or *RASSF1A *were considered informative cases for further comparisons. The MSP results were challenged by cytological analysis during the post-surgical monitoring: 5/12 patients showed positive cytology at the time of cell collection to MSP analysis, but only one of these exhibited *RARB *hypermethylation in the same washout cells. MSP analysis results of *RASSF1A *gene in 7 informative patients were discordant because none of the 4 recurrent cases by cytological analysis showed the epigenetic marker; contrarily, *RASSF1A *hypermethylation was detected in 2 of the 3 non-recurrent cases. Table 5 exhibits the sensitivity and specificity of MSP analysis in relation to the gold-standard cytological evaluation for tumor recurrence detection.

**Table 5:** Accuracy estimatives from MSP for *RARB *and *RASSF1A *genes observed in primary and/or previous recurrence of urinary bladder carcinoma and respective washout.

**Gene**	**Accuracy patterns (%)**				
	**Sensitivity**	**Specificity**	**PPV**	**NPV**	**p^(1)^**
**Sediment of washout cells**					

RARB	1/6 (16.7)	13/14 (92.8)	1/2 (50.0)	13/18 (72.2)	0.521
*RASSF1A*	0/4 (0.00)	5/10 (50.0)	0/5 (0.00)	5/9 (55.5)	0.220

**Fixed and paraffin-embedded bladder cancer tissue**					

RARB	5/6 (83.3)	7/14 (50.0)	5/12 (41.6)	7/8 (87.5)	0.324
*RASSF1A*	4/4 (100.0)	4/7 (57.1)	4/7 (57.1)	4/4 (100.0)	0.193

Cytology results were used as reference to detect the recurrence.

(1) Fisher's Exact test (α = 0.05); PPV – positive predictive value; NPV – negative predictive value

## Discussion

Epigenetic alterations are a hallmark of human cancer. In particular, DNA hypermethylation is a common mechanism for inactivating tumor-suppressor and other cancer genes in tumor cells [48]. The aberrant methylation patterns have been used as targets for the detection of tumor cells in clinical specimens such as tissue biopsies or body fluids [49].

In our MSP analysis performed on DNA obtained from fresh tumor samples, a hypermethylated pattern predominated for *CDH1 *and *SFN *genes. Commonly, a large spectrum of hypermethylation frequencies has been reported for several genes in bladder cancer. For example, *CDH1 *gene methylation frequencies range from 9.5% to 84% [15,22,25,27,30,37-40], independently of histological classification. We detected *CDH1 *hypermethylation frequency of 100% in bladder UCs, squamous cell carcinomas and adenocarcinomas samples, as well as in normal adjacent urinary bladder tissue and in exfoliated urothelial cells from cancer-free controls. Similarly, the *SFN *gene was also hypermethylated in these samples. Costa et al. [50] also detected high frequencies of *CDH1 *methylation in clear cell renal carcinomas and normal renal tissues (82.7% and 87.1%, respectively); in addition, *SFN *was hypermethylated in 100% of normal and tumoral renal tissues analyzed.

Interpretation of differential DNA-methylation patterns in cancer has proven difficult, in part because the functional consequences depend on the genomic region involved, the specific CpG dinucleotides, and the inter- and intratumoral heterogeneity. Apart from this, methodological issues such as the different primer sets interrogating methylation at distinct CpG dinucleotides of a specific promoter region could explain the range of frequencies reported in the literature. In our study, the protocol used (which included the addition of urea in the DNA modification step in order to improve the efficiency of unmethylated cytosine conversion [42]) and the *CDH1 *gene analysis based on the nested-PCR approach may have contributed to the high methylation prevalence observed.

The dynamic nature of epigenetic alterations is partially due to polymorphisms in some methyl group metabolism genes [51,52] and in genes coding for proteins that mediate these changes (DNA methyltransferases, methyl-CpG-binding domain proteins) [53]. In addition, genomic profiles of DNA methylation are also influenced by aging [38,54], dietary intake [55,56] and environmental exposure [53,57,58]. In this context, DNA methylation heterogeneous patterns should be expected and already detected for some genomic regions as reported by Eckkhardt et al. [59]. These authors have found that 30.2% of investigated loci on chromosomes 6, 20, and 22 exhibit heterogeneity of methylation status, mainly due to the mosaic patterns in the studied tissue. Furthermore, 10% of the analyzed regions showed tissue-specific differences in DNA methylation.

We observed hypermethylated *CDH1*, *SFN*, and *RARB *genes in the normal-adjacent tissue of urinary bladder tumor. Aberrant methylation patterns have been associated with chronic inflammation [60,61], viral infection [62] and aging [63]. Smith and Pereira-Smith [64] have previously reported that epigenetic alterations are involved in both the etiology and consequences of aging. Thus, hypermethylation in normal tissue as detected in the present study agrees with the results previously found by Bornman et al. [38], who observed a similar pattern for *CDH1 *in normal bladder tissue from patients older than 70 years. Furthermore, aberrant methylation patterns of the *CDH1 *promoter region were also described in pre-adenoma stages of colorectal cancer, in hyperplasic polyps [65,66] and in ulcerative colitis (a chronic inflammatory condition of the large intestine that predisposes to cancer) [60,67]. During breast cancer progression, *CDH1 *gene methylation occurs in about 30% of the ductal carcinomas *in situ*, with a significant increase in invasive and metastatic lesions [68]. Moreover, this gene has been also found methylated in pre-malignant and invasive bladder cancers. In mammary tissue, *SFN *is usually unmethylated in normal epithelium, but methylated in atypical hyperplasias, intraductal papillomas, ductal *in situ *carcinomas, infiltrating carcinomas and in stromal cells [69,70]. *SFN *and *CDH1 *methylation have been reported in peripheral blood cells [70] as well as in infiltrating leukocytes in breast cancer [71]. Overall, these observations suggest that both genes, *CDH1 *and *SFN*, are not effective biomarkers for MSP analysis in bladder cancer.

The MSP analysis of *RARB *and *RASSF1A *genes showed respective hypermethylation of 82.9% and 24.4% in 49 UCs analyzed. Investigating the methylation at the same CpG dinucleotides, Maruyama et al. [22], and Catto et al. [25], found 15% and 24% hypermethylation for *RARB*, and 35% and 54% for *RASSF1A*, respectively. In order to verify the specificity of *RARB *and *RASSF1A *hypermethylation in relation to malignant phenotype in bladder tissue, we evaluated these genes in exfoliated urothelial cells from patients without cancer (control group): *RARB *and *RASSF1A *hypermethylation were detected in 8.3% and 33.3%, respectively. The comparison of these data revealed that *RARB *hypermethylation provides better diagnostic coverage and specificity than *RASSF1A *hypermethylation. However, the hypermethylated pattern of these genes in normal adjacent tissue in matched samples, especially for the *RARB *gene, was an unexpected finding. In this context, we could hypothesize that molecular alterations precede morphological changes in the exposed urinary bladder epithelium, since patients with bladder neoplasia frequently show genetic instability on apparently normal mucosa besides alterations of surrounding tissue [72]. Aberrant methylation patterns appear to reflect a pre-malignant characteristic of the urinary bladder mainly because UC is a neoplasia with multifocal lesions and elevated recurrence indices [73], thus corroborating the hypothesis that epigenetic alterations in cancer may preexist in morphologically normal cells [74].

Thus, genetic and epigenetic alterations may be present before cancer detection by imaging or traditional pathology investigations. Therefore, molecular tests that target these alterations have conceptual advantages for the successful early detection of neoplasias [48]. DNA represents an ideal substrate for molecular detection because it is robust, survives adverse conditions that many clinical specimens undergo and, can be readily amplified by powerful PCR-based approaches [75]. Tiny amounts of DNA from early pre-neoplastic lesions or small cancers can be used to permit the sensitive detection of one cancer cell in a background of hundreds of normal cells.

Hence, the predictive value of MSP in identifying tumor cells in washing sediments was evaluated in bladder cancer patients under post-surgical monitoring to detect tumor recurrence. Positive cytology was found in 33.3% of patients with urinary bladder tumor history. The hypermethylation patterns of *RARB *and *RASSF1A *genes observed in cells obtained from urinary bladder washing sediments were not concordant: some hypermethylated cases in tumor tissue and recurrence by cytological analysis did not show this marker in the same exfoliated cells. Contrarily, in four cases, the cells taken from urinary bladder washings exhibiting hypermethylation for these genes did not match the hypermethylation of the correspondent TCC. The heterogeneity of the intra- and intertumoral methylation patterns could partially explain these discrepancies. Thus, hypermethylation could already be present in the urinary bladder epithelium of cancer patients but not necessarily in cells exfoliated from the urinary bladder of cancer-free patients. *RARB *hypermethylation confirmed the presence of tumor cells in only 1 out of 5 recurrent cases and was absent in all cases showing negative cytology. Importantly, for the eight patients whose tumors did not present *RARB *methylation, the paired cell washing sediments DNA were also negative for methylation. This finding corroborates the idea that the methylation pattern of this gene is specific for tumor cells. *RASSF1A *gene MSP analysis in washout cells showed discordant results since its hypermethylation was not detected in 4 recurrent cases, although 2 negative cases for tumor cells using cytology showed this tumor tag, which suggest that these patients are under high risk for tumor recurrence. Some studies using promoter hypermethylation identified in tumor DNA as a target for cancer detection in the correspondent urine sample have shown sensitivities ranging from 49% to 91% [15,26,30,75]. Recently, Yu et al. [76] included the *RASSF1A *in an 11-gene set to assessment of DNA methylation in urine sediments for sensitive/specific detection of bladder cancer. Although two studies have reported that the overall methylation sensitivity was significantly higher than cytology [15,77], several factors may contribute to the lower sensitivities of MSP analysis in cells from urinary bladder fluids including the incomplete diagnostic coverage of selected gene sets, limited quantity of cells sampled, and the intrinsic heterogeneity of methylation patterns in the exposed epithelium, among others.

## Conclusion

In the literature, no single gene was found to be consistently methylated in most bladder tumors. Thus, panels of genes that are methylated in urinary bladder cancer have been investigated to define methylation profiles associated with urinary bladder cancer diagnosis, prognosis and early recurrence detection. DNA hypermethylation of *CDH1 *and *SFN *genes was detected indistinctly among urinary bladder tumoral and normal tissues as well as in urinary bladder exfoliated cells, suggesting that these epigenetic features do not satisfy enough specificity criteria for use as prognostic or early detection markers. The methylation of *RARB *and *RASSF1A *genes appears to be an initial event in urinary bladder carcinogenesis maintained during tumor progression and should be included in the panels of differentially methylated genes in urinary bladder cancer in order to maximize the diagnostic coverage of epigenetic markers.

## Abbreviations

*CDH1*: cadherin 1, type 1, E-cadherin [epithelial] gene; *LOH*: loss of heterozygosity; *MSP*: methylation-specific PCR; *RARB*: retinoic acid receptor, beta gene; *RASSF1A*: Ras association (RalGDS/AF-6) domain family 1 gene; *SFN*: stratifin, also know as 14-3-3σ gene; *TCC*: transitional cell carcinoma; TSG: tumor suppressor gene; *UC*: urothelial carcinoma.

## Competing interests

The authors declare that they have no competing interests.

## Authors' contributions

PDN assisted in the study design, in defining the casuistic used, in collecting fresh bladder samples, carrying out the molecular genetic studies and performing the statistical analysis. CAR assisted in the study design, in defining the casuistic used, carrying out the molecular genetic studies and performing the statistical analysis. FPF carried out the molecular genetic studies. JLVC performed the histopathological analysis and classified all fresh bladder samples. MLCSO carried out the cytological analysis of all urinary bladder washings. JG collected urinary bladder washing samples. DMFS was responsible for the study coordination, assisted in the design of the study and in defining the casuistic used. All authors helped to draft the manuscript, and to read and approve the final version.

## Pre-publication history

The pre-publication history for this paper can be accessed here:


